# Gallbladder Stone Prevalence and Related Factors in Predialysis Chronic Kidney Disease Patients

**DOI:** 10.5152/tjg.2022.22350

**Published:** 2022-09-01

**Authors:** Üstün Yılmaz, Ayça İnci, Ercan Özcan, Semih Gül, Hatice Gizem Berber, Firdevs Pelin Eşkin, Yasin Şahintürk, Ayhan Hilmi Çekin

**Affiliations:** 1 Department of Nephrology, University of Health Sciences Antalya Training and Research Hospital, Antalya, Turkey; 2 Department of Internal Medicine, University of Health Sciences Antalya Training and Research Hospital, Antalya, Turkey; 3 Department of Gastroenterology, University of Health Sciences Antalya Training and Research Hospital, Antalya, Turkey

**Keywords:** Chronic kidney disease, gallbladder stone, predialysis, associated factor

## Abstract

**Background:**

The aim of this study was to determine the prevalence and factors associated with gallbladder stone in patients with predialysis chronic kidney disease.

**Methods:**

This cross-sectional study retrospectively examined follow-up data of patients with chronic kidney disease between January 2015 and December 2020 at the Health Sciences University Antalya Training and Research Hospital who had undergone abdominal ultrasonography for any reason. Patients with gallbladder stone on abdominal ultrasonography and history of cholecystectomy due to gallbladder stone were identified as the gallstone group. The prevalence of gallbladder stone was determined according to disease stage. Patients with and without gallbladder stone were compared in terms of demographic and clinical characteristics and laboratory parameters that may be associated with the development of gallbladder stone.

**Results:**

A total of 511 patients had chronic kidney disease (stages 3, 4, and 5 in 303 [59.3%], 176 [34.4%], and 32 [6.3%], respectively). The gallstone prevalence rates were 25.1%, 30.1%, and 46.9% in stage 3, 4, and 5 chronic kidney disease, respectively, and that in all the patients was 28.2% (95% CI: 24.3-32.3, *P* = .026). Logistic regression analysis revealed that increased age (odds ratio: 1.045; 95% CI: 1.027-1.063, *P* < .001) and decreased estimated glomerular filtration rate (odds ratio: 0.974; 95% CI: 0.956-0.991, *P* = .004) were associated with gallbladder stone.

**Conclusion:**

The prevalence of gallbladder stone was high in the predialysis patients with chronic kidney disease and increased with increasing disease stage. High age and low estimated glomerular filtration rate were associated with gallbladder stone formation.

**Main Points**

- Our study aimed to determine the frequency of gallbladder stone (GBS) in patients followed up for predialysis chronic kidney disease (CKD) and the factors that may be related to GBS formation.
- Our findings show that the prevalence of GBS was higher in patients with predialysis CKD than in the general population without any renal disease.
- We observed that the prevalence increased as the CKD stage increased.
- Older age and low estimated glomerular filtration rate (eGFR) were found to be factors associated with GBS formation.
- This study will stimulate broader studies in the future that will specifically examine the relationship between eGFR reduction and the occurrence of GBS.

## Introduction

Gallbladder stones (GBS) are an important health problem worldwide. It can cause many complications with high mortality and morbidity, such as cholecystitis, cholangitis, jaundice, or pancreatitis. These conditions also increase health costs. The prevalence of GBS in the community is generally reported to be 10%-15%.^1-3^ In a study conducted in Turkey with 2188 cases from the general population, gallstones were found in 7% of women and 3.5% of men.^4^ Race, genetic factors, female sex, age, body mass index (BMI), alcohol, smoking, drug (oral contraceptive, thiazide diuretics, octreotide, ceftriaxone, etc.) use, ileal diseases, hemolytic anemias, diabetes mellitus (DM), and hyperlipidemia have been reported to be associated with GBS.^1,5-7^

Although GBS is a common condition in Western countries, data on the incidence of end-stage renal disease (ESRD) are limited. The occurrence of GBS in patients fed with low-protein diets suggests that GBS formation is affected by dietary protein content.^8,9^ This condition is quite common in patients with advanced chronic kidney disease (CKD). In some studies, the prevalence of GBS has been shown to increase in patients undergoing hemodialysis (HD) treatment for ESRD.^10-13^ In a study by Kazama et al^14^, the prevalence of GBS increased with the progression of the disease stage in patients with predialysis CKD not undergoing HD (CKD1: 7.7%; CKD2: 15.4%; CKD3: 19.0%; CKD4: 20.8%; and CKD5: 21.3%). However, other studies reported that patients with CKD showed a similar prevalence of GBS formation to the general population.^15-17^ This information is insufficient to say that CKD is a risk factor for GBS formation. Although many studies in the literature have shown increased prevalence rates of GBS in HD patients, few studies have evaluated the prevalence of GBS in patients with predialysis CKD. This suggests that new studies are needed to investigate the prevalence of GBS in patients with predialysis CKD and to identify risk factors.

Our aim in this study was to determine the frequency of GBS in patients followed up for predialysis CKD and the factors that may be related to GBS formation.

## Material and Methods

This cross-sectional study was conducted by retrospectively examining the follow-up data of patients diagnosed as having CKD between January 2015 and December 2020 at the Health Sciences University Antalya Training and Research Hospital. The study data were collected by the authors after an 8-month detailed analysis of the patient follow-up records, taking into account the confidentiality of the patient data. A standard study form was prepared, all patient records were analyzed with the same algorithm, and then data were collected and recorded. This study was approved by the ethics committee of the Scientific Research Ethics Committee of Health Sciences University Antalya Training and Research Hospital and was conducted in accordance with the ethical standards defined in the 1964 Declaration of Helsinki. Since our study was retrospective, informed consent was not obtained from the patients.

Patients who were diagnosed as having CKD in Health Sciences University Antalya Training and Research Hospital Nephrology Clinic and had follow-up records were included in the study. In the sample, patients aged >18 years who had undergone abdominal ultrasonography for any reason in the past were selected. Chronic kidney disease patients with GBS on abdominal ultrasonography and history of cholecystectomy due to GBS were identified as the gallstone group. Patients with no GBS and normal gallbladder on abdominal ultrasonography were determined as the non-gallstone group. The CKD disease stage of the patients was determined and divided into 3 groups according to the estimated glomerular filtration rate (eGFR), calculated using the formula developed by the CKD Epidemiology Cooperation.^18^ According to this calculation, the eGFR according to CKD disease stage were as follows: stage 3, 30-59 mL/min/1.73 m^2^; stage 4, 15-29 mL/min/1.73 m^2^; and stage 5, <15 mL/min/1.73 m^2^.^19^ Chronic kidney disease was defined by Kidney Disease Quality Outcome Initiative as an eGFR <60 mL/min/1.73 m^2^ for 3 months or more, irrespective of the cause, and it was reported that CKD-related signs and symptoms started after this stage.^20^ Therefore, patients with eGFR <60 mL/min/1.73 m^2^ were included in the study. Demographic and laboratory parameters were recorded to evaluate the CKD patients with and without GBS in terms of factors that may be associated with GBS. Patients who received a kidney transplant and those with CKD who were aged <18 years were excluded from the study. In addition, CKD patients with liver cirrhosis and Crohn’s disease were excluded from the study because of risk factors of GBS.

The following demographic data of the patients were examined: age, sex, BMI, educational status, place-of-residence status, alcohol and smoking status, CKD duration, eGFR, DM, hyperlipidemia, and drug use with the possibility of GBS (oral contraceptive, thiazide diuretics, octreotide, and ceftriaxone). As laboratory data, high-density lipoprotein (HDL), low-density lipoprotein (LDL), total cholesterol, triglyceride, total bilirubin, direct bilirubin, alanine aminotransferase (ALT), gamma-glutamyl transferase (GGT), alkaline phosphatase (ALP), uric acid, calcium, phosphorus, parathyroid hormone (PTH), albumin, and total protein levels were also examined.

## Statistical Analysis

Statistical analysis was performed using International Business Machines Statistical Package for the Social Sciences Statistics for Windows, Version 23.0 (IBM Corp., Armonk, NY). Normality assumptions were controlled by the Shapiro–Wilk test. Descriptive analyses were presented using mean ± standard deviation, median (interquartile range), or frequency (percentages), where appropriate. Categorical data were analyzed with Pearson’s chi-square and Fisher’s exact tests. The Mann–Whitney *U* test and an unpaired *t*-test were used to analyze non-normally and normally distributed numerical data, respectively. A multivariable logistic regression analysis was performed to determine independent risk factors associated with gallbladder stone. Variables with *P* < .2 in the univariate analyses were further tested in the multivariable model. Odds ratios (OR) with corresponding 95% CI were reported. *P* < .05 was considered statistically significant.

## Results

A total of 511 patients with CKD, including 240 women (47%) and 271 men (53%), were included in the study. When the included patients were examined according to their CKD stages, 303 (59.3%), 176 (34.4%), and 32 (6.3%) were found to have stage 3, 4, and 5 predialysis CKD. The mean age of the patients was 62.9 ± 14.2 years, and the mean BMI was 27.1 ± 3.9 kg/m^2^. Considering educational status, 442 patients (86.5%) were pre-college graduates and 69 (13.5%) were college graduates. According to place-of-residence status, 24 patients (4.7%) lived in villages and 487 (95.3%) lived in cities. Nine patients (1.8%) used alcohol and 186 (36.4%) smoked. The CKD duration was 5 years (range: 3-6 years). The mean eGFR was 33 mL/min/1.73 m**^2^** (range: 23-44 mL/min/1.73 m^2^). Diabetes mellitus and hyperlipidemia were found to be comorbidities in 208 (40.8%) and 167 (32.7%) patients, respectively.

In 39 patients (7.6%), drug use that could cause GBS was observed. The values of the laboratory parameters were as follows: HDL, 50 mg/dL (40-53 mg/dL); LDL, 117 mg/dL (93-140 mg/dL); total cholesterol, 196 mg/dL (168-221 mg/dL); triglyceride, 150 mg/dL (100-200 mg/dL); total bilirubin, 0.5 mg/dL (0.4-0.7 mg/dL); direct bilirubin, 0.1 (0.1-0.1 mg/dL); ALT, 15 U/L (10-20 U/L); GGT, 27 U/L (18-40 U/L); ALP, 70 U/L (56-91 U/L); uric acid, 6.8 ± 1.8 mg/dL; calcium, 9.4 ± 0.7 mg/dL; phosphorus, 3.7 ± 0.8 mg/dL; PTH, 90 pg/mL (55-142 pg/mL); albumin, 4.2 g/L (3.8-4.5 g/L); and total protein, 7.2 g/L (6.7-7.6 g/L) (Table 1).

**Figure 1.**
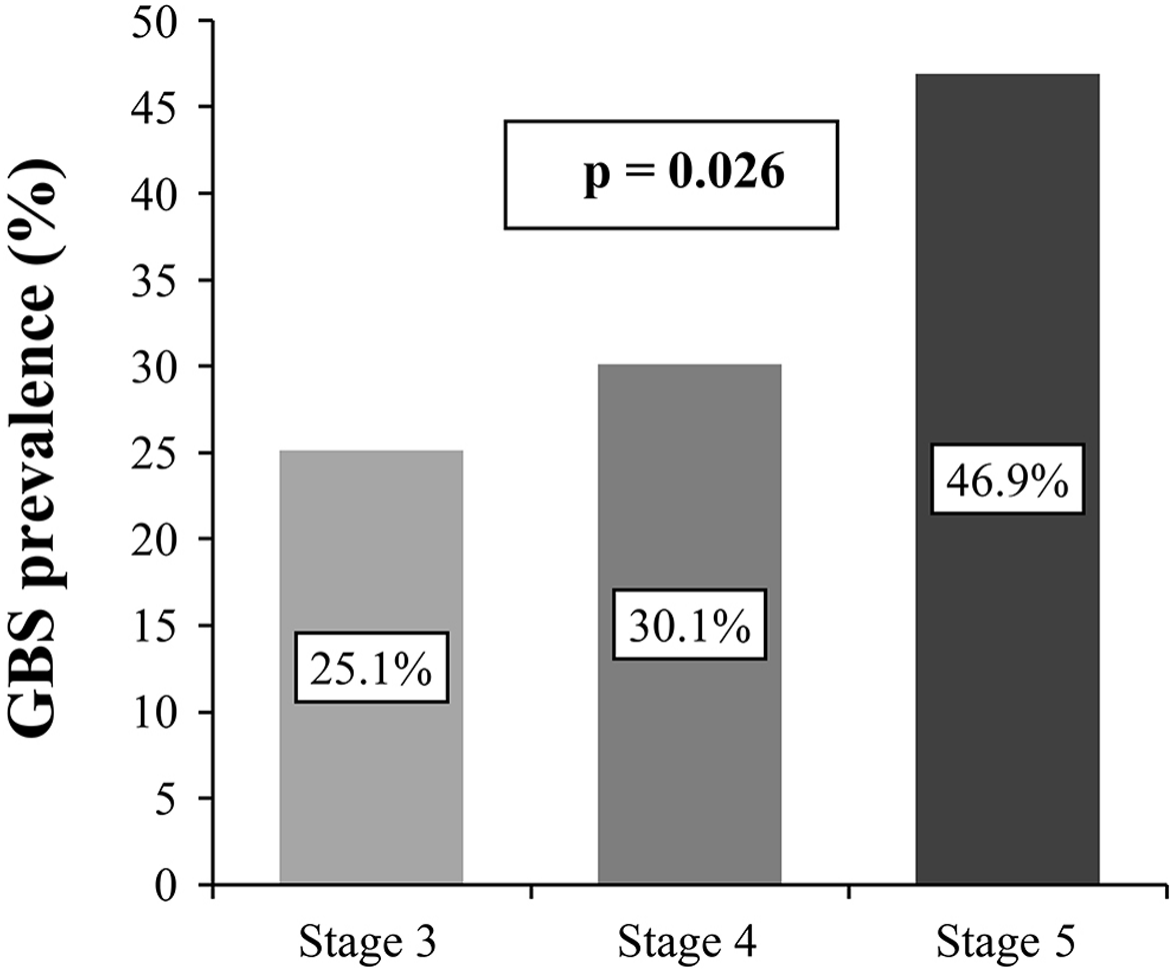
Prevalence of gallbladder stone (GBS) according to chronic kidney disease stage (95% CI: 24.3-32.3; *P* = .026, Pearson’s chi-square test).

The prevalence of GBS in the whole sample was 28.2%. According to CKD stage, the prevalence rates of GBS were 25.1%, 30.1%, and 46.9% in stages 3, 4, and 5 CKD, respectively. As the CKD stage increased, the prevalence of GBS increased (95% CI: 24.3-32.3, *P* = .026 by Pearson’s chi-square test) (Figure 1).

Chronic kidney disease patients with and without GBS were compared according to various variables. We found that age was older (*P* < .001) and eGFR level was lower (*P* < .001) in the patients with GBS. Sex (*P* = .389), BMI (*P* = .965), educational status (*P* = .678), place-of-residence status (*P* = .723), alcohol use (*P* = .999), smoking (*P* = .190), CKD duration (*P* = .414), DM (*P* = .082), hyperlipidemia (*P* = .381), and use of drugs that cause GBS (*P* = .138) showed no significant differences between the groups. When compared, the following laboratory parameters did not significantly differ between the groups: HDL (*P* = .071), LDL (*P* = .891), total cholesterol (*P* = .576), triglyceride (*P* = .327), total bilirubin (*P* = .800), direct bilirubin (*P* = .831), ALT (*P* = .546), GGT (*P* = .850), uric acid (*P* = .118), calcium (*P* = .075), phosphorus (*P* = .309), PTH (*P* = .309), and total protein (*P* = .208). The mean ALP level was higher (*P* = .037), and the mean albumin level was lower in those with GBS (*P* < .001) (Table 2).

Logistic regression analysis was performed to identify factors associated with GBS. The relationships between the occurrence of GBS with increasing age (OR: 1.045; 95% CI: 1.027-1.063, *P* < .001) and decreased eGFR (OR: 0.974; 95% CI: 0.956-0.991, *P* = .004) were determined. Smoking (OR: 1.045; 95% CI: 0.638-1.71, *P* = .862), DM (OR: 1.158; 95% CI: 0.746-1.799, *P* = .513), use of drugs that cause GBS (OR: 1.712; 95% CI: 0.812-3.609, *P* = .158), uric acid level (OR: 1.117; 95% CI: 0.996-1.253, *P* = .059), HDL level (OR: 0.989; 95% CI: 0.971-1.007, *P* = .211), ALP level (OR: 1.004; 95% CI: 0.999-1.008, *P* = .114), calcium level (OR: 0.994; 95% CI: 0.664-1.49, *P* = .977), albumin level (OR: 0.756; 95% CI: 0.458-1.247, *P* = .274) were not associated with GBS formation (Table 3).

## Discussion

In our study, the prevalence of GBS was found to be higher in patients with predialysis CKD as compared with that in the general population worldwide and in Turkey.^1-4^ When examined according to the disease stage, the prevalence of GBS increased with increasing CKD stage. This suggests that the disease may pose a risk in terms of GBS formation from its early stages. In a previous study on this topic, the prevalence of GBS in predialysis patients with CKD was 7.7% in those with stage 1 CKD and 21.3% in those with stage 5 CKD. In the same study, the prevalence was 5.9% in the healthy control group.^14^ In another study by Vecchi et al^21^, predialysis patients under conservative treatment were compared with the general population in terms of GBS prevalence. In the study, while the GBS prevalence in patients with predialysis CKD was 22%, it was 11.5% in the general population. When the essence and purpose of these similar studies were examined, their findings support those of our study in that the prevalence of GBS was higher in patients with CKD than in the general population and increased with increasing CKD stage. It is of note that there were differences in numerical prevalence between our study and similar studies reported previously, which we think may be due to the different ethnicities of the study population.

Publications have reported that age is an important factor in the occurrence of GBS in the general population. A previous study on the subject reported that the prevalence of GBS may be 10 times higher in people aged ≥40 years.^22^ With aging, the probability of gallstone formation increases as a result of decreased synthesis of 7α-hydroxylase, the rate-limiting enzyme for the regulation of bile acid pool size and conversion of cholesterol to bile acids, increased bile cholesterol saturation, and decreased gallbladder emptying motility.^2,23^ Some studies in patients with CKD, similar to those reported in the general population, tended to show a higher prevalence of GBS in older patients.^14,24,25^ In contrast to these studies, other studies reported no significant relationship between age and GBS formation in comparisons between patients with CKD and controls.^10,21^ In our study, among the factors, age was associated with GBS.

The prevalence of GBS in CKD patients has been reported to be higher than that in the general population.^21,24^ On the contrary, other studies reported that the prevalence of GBS in HD patients was similar to that in the general population.^15-17^ Different from the previous studies, our present study, regardless of CKD stage and the presence of CKD disease itself, identified a significant relationship between decreased eGFR and GBS formation. To our knowledge, no study has examined eGFR among the factors that may be associated with GBS. Estimated glomerular filtration rate reduction may have increased the occurrence of GBS by causing patients to receive diets for severe CKD and to be fed low-protein diets. Consistent with this finding, studies have reported higher prevalence rates of GBS formation in CKD patients fed low-protein diets.^9^ In addition, metabolic disorders due to advanced kidney disease along with decreased eGFR may have caused changes in bile composition. As eGFR decline was found as among the factors associated with GBS in our present study but the reason could not be explained, future studies should focus on investigating the potential value of eGFR as a parameter indicating GBS formation.

In the logistic regression analysis performed to identify factors associated with GBS, age and eGFR were found to be significantly associated with the occurrence of GBS in patients with predialysis CKD. However, causes such as DM, hyperlipidemia, and female gender, which are all risk factors for GBS in the general population, were not found as risk factors in our study population. This may be due to the comparison among patients with CKD without including a healthy population for comparison. In addition, the adverse effects of eGFR reduction alone may have led to the formation of GBS in patients with predialysis CKD through a yet unknown mechanism. Considering these situations, more comprehensive studies are needed on this subject.

One of the limitations of our study is its retrospective design, in which we had limited control over the quality of the patient data used for the analysis. For example, ultrasonographic examinations were not aimed at targeting gallbladder pathologies. In addition, we could not determine the types of gallstone (bilirubin, cholesterol, or mixed) and whether a blood transfusion was performed, as it was not in the patient records. Another limitation is that the number of patients with stage 5 CKD before dialysis was lower than those of patients with other stages of CKD. To minimize biases that these limitations may cause, more studies on the subject are needed.

## Conclusions

Our findings show that the prevalence of GBS was higher in the patients with predialysis CKD than in the general population without renal disease. In addition, we observed that the prevalence increased as the CKD stage increased. High age and low eGFR were found to be factors associated with GBS formation. We hope that this study will stimulate broader studies in the future that will specifically examine the relationship between eGFR reduction and the occurrence of GBS.

## Figures and Tables

**Table 1.** Demographic and Clinical Characteristics and Laboratory Parameters of the Patients with Chronic Kidney Disease

Variables	n = 511, mean ± SD/n (%)/median (IQR)
Age (years)	62.9 ± 14.2
Sex	
Female	240 (47%)
Male	271(53%)
BMI (kg/m**2**)	27.1 ± 3.9
Educational status	
Pre-college	442 (86.5%)
College graduate	69 (13.5%)
Place-of-residence status	
Village	24 (4.7%)
City	487 (95.3%)
Alcohol	9 (1.8%)
Smoking	186 (36.4%)
CKD duration (years)	5 (3-6)
eGFR (mL/min/1.73 m**2**)	33 (23-44)
Stage	
3	303 (59.3%)
4	176 (34.4%)
5	32 (6.3%)
Comorbidities	
DM	208 (40.8%)
Hyperlipidemia	167 (32.7%)
Use of drugs that cause GBS^a^	39 (7.6%)
HDL (mg/dL)	50 (40-53)
LDL (mg/dL)	117 (93-140)
Total cholesterol (mg/dL)	196 (168-221)
Triglyceride (mg/dL)	150 (100-200)
Total bilirubin (mg/dL)	0.5 (0.4-0.7)
Direct bilirubin (mg/dL)	0.1 (0,1-0,1)
ALT (U/L)	15 (10-20)
GGT (U/L)	27 (18-40)
ALP (U/L)	70 (56-91)
Uric acid (mg/dL)	6.8 ± 1.8
Calcium (mg/dL)	9.4 ± 0.7
Phosphorus (mg/dL)	3.7 ± 0.8
PTH (pg/mL)	90 (55-142)
Albumin (g/L)	4.2 (3.8-4.5)
Total protein (g/L)	7.2 (6.7-7.6)

^a^Oral contraceptive, thiazide diuretics, octreotide, and ceftriaxone.

BMI, body mass index; CKD, chronic kidney disease; eGFR, estimated glomerular filtration rate; DM, diabetes mellitus; GBS, gallbladder stone; HDL, high-density lipoprotein; LDL, low-density lipoprotein; ALT, alanine aminotransferase; GGT, gamma-glutamyl transferase; ALP, alkaline phosphatase; PTH, parathyroid hormone; SD, standard deviation; IQR, interquartile range.

**Table 2.** Comparison of Demographic and Clinical Characteristics and Laboratory Parameters According to Gallbladder Stone Status of the Patients with Chronic Kidney Disease

Variables	With Gallbladder Stone (n = 144)	Without Gallbladder Stone (n = 367)	*P*
Age (years)	69 ± 11.6	60.5 ± 14.5	<.001
Sex			
Female	72 (50%)	168 (45.8%)	.389
Male	72 (50%)	199 (54.2%)	
BMI (kg/m**2**)	27.1 ± 3.6	27.1 ± 4	.965
Educational status			
Pre-college	126 (87.5%)	316 (86.1%)	.678
College graduate	18 (12.5%)	51 (13.9%)	
Living place status			
Village	6 (4.2%)	18 (4.9%)	.723
City	138 (95.8%)	349 (95.1%)	
Alcohol	2 (1.4%)	7 (1.9%)	.999
Smoking	46 (31.9%)	140 (38.1%)	.190
CKD duration (years)	5 (3-7)	5 (3-6)	.414
eGFR (mL/min/1.73 m**2**)	31.5 (20-38)	34 (24-47)	<.001
Comorbidities			
DM	67 (46.9%)	141 (38.4%)	.082
Hyperlipidemia	51 (35.7%)	116 (31.6%)	.381
Use of drugs that cause GBS^a^	15 (10.4%)	24 (6.5%)	.138
HDL (mg/dL)	46 (40-51.5)	50 (40-53)	.071
LDL (mg/dL)	120 (89-140)	115 (100-140)	.891
Total cholesterol (mg/dL)	197.5 (160-220.5)	192 (170-221)	.576
Triglyceride (mg/dL)	160 (110-200)	150 (100-200)	.327
Total bilirubin (mg/dL)	0.5 (0.4-0.7)	0.5 (0.4-0.7)	.800
Direct bilirubin (mg/dL)	0.1 (0.1-0.1)	0.1 (0.1-0.1)	.831
ALT (U/L)	14 (10-20)	15 (10-20)	.546
GGT (U/L)	25.5 (17-40)	28 (18-40)	.850
ALP (U/L)	72 (60-100)	70 (51-90)	**.037**
Uric acid (mg/dL)	7 ± 1.9	6.8 ± 1.8	.118
Calcium (mg/dL)	9.3 ± 0.7	9.4 ± 0.7	.075
Phosphorus (mg/dL)	3.7 ± 0.8	3.7 ± 0.7	.309
PTH (pg/mL)	90 (60.5-161)	90 (51-141)	.309
Albumin (g/L)	4 (3.7-4.3)	4.2 (3.9-4.5)	<.001
Total protein (g/L)	7 (6.6-7.5)	7.2 (6.7-7.6)	.208

Data are presented as mean ± SD, median (IQR), or n (%). Unpaired *t*-test, Mann–Whitney *U* test, Pearson’s chi-square test, and Fisher’s exact test were used in the analyses.

Statistically significant findings are indicated in bold.

^a^Oral contraceptive, thiazide diuretics, octreotide, and ceftriaxone.

BMI, body mass index; CKD, chronic kidney disease; eGFR, estimated glomerular filtration rate; DM, diabetes mellitus; GBS, gallbladder stone; HDL, high-density lipoprotein; LDL, low-density lipoprotein; ALT, alanine aminotransferase; GGT, gamma-glutamyl transferase; ALP, alkaline phosphatase; PTH, parathyroid hormone; SD, standard deviation; IQR, interquartile range.

**Table 3.** Logistic Regression Analysis to Determine Factors Associated with Gallbladder Stone

Variable	OR (95% CI)	*P*
Age (years)	1.045 (1.027-1.063)	<.001
Smoking	1.045 (0.638-1.71)	.862
DM	1.158 (0.746-1.799)	.513
Use of drugs that cause GBS	1.712 (0.812-3.609)	.158
eGFR (mL/min/1.73 m**2**)	0.974 (0.956-0.991)	**.004**
Uric acid (mg/dL)	1.117 (0.996-1.253)	.059
HDL (mg/dL)	0.989 (0.971-1.007)	.211
ALP (U/L)	1.004 (0.999-1.008)	.114
Calcium (mg/dL)	0.994 (0.664-1.49)	.977
Albumin (g/L)	0.756 (0.458-1.247)	.274

The variables with *P* < .2 in the univariate analysis were included in the multivariable model.

Statistically significant findings are indicated in bold.

eGFR, estimated glomerular filtration rate; DM, diabetes mellitus; GBS, ­gallbladder stone; HDL, high-density lipoprotein; ALP, alkaline phosphatase.
